# Multi-OCT-SelfNet: integrating self-supervised learning with multi-source data fusion for enhanced multi-class retinal disease classification

**DOI:** 10.3389/fsysb.2026.1717398

**Published:** 2026-06-01

**Authors:** Fatema E. Jannat, Sina Gholami, Jennifer I. Lim, Theodore Leng, Minhaj Nur Alam, Hamed Tabkhi

**Affiliations:** 1 Department of Electrical and Computer Engineering, University of North Carolina at Charlotte, Charlotte, NC, United States; 2 University of Illinois at Chicago, Chicago, IL, United States; 3 Stanford University School of Medicine, Stanford, CA, United States

**Keywords:** AI, data fusion, OCT, retinal disease classification, self-supervised learning, SwinV2, transfer learning, transformer

## Abstract

Acquiring large and diverse medical imaging datasets remains challenging because of privacy, annotation cost, and institutional variability. This limitation can reduce the generalization ability of deep learning models, particularly when they are trained on small or domain-specific retinal datasets. To address this issue, we propose Multi-OCT-SelfNet, a self-supervised framework based on a SwinV2 transformer backbone for multi-class retinal disease classification from optical coherence tomography (OCT) images. The framework combines multi-source OCT datasets during masked autoencoder-based self-supervised pre-training to learn transferable image representations, followed by supervised fine-tuning on individual downstream datasets. We evaluated Multi-OCT-SelfNet across three benchmark OCT datasets (DS1, DS2, and DS3) and compared its performance with two baselines: ResNet-50 and traditional SwinV2 trained without the proposed self-supervised multi-source pre-training strategy. In on-domain evaluation, Multi-OCT-SelfNet-SwinV2 achieved AUC-ROC scores of 0.97 on DS1, 0.97 on DS2, and 0.89 on DS3, demonstrating competitive or improved performance compared with both baselines. The advantage of the proposed framework was more evident in cross-dataset evaluation, especially for smaller datasets. When trained on DS2 and tested on DS3, Multi-OCT-SelfNet-SwinV2 improved AUC-ROC from 0.59 with ResNet-50 and 0.61 with traditional SwinV2 to 0.90. Similarly, when trained on DS3 and tested on DS2, the proposed model achieved an AUC-ROC of 0.94, compared with 0.60 for ResNet-50 and 0.81 for traditional SwinV2. Under limited-data settings using only 50% of the training samples, Multi-OCT-SelfNet-SwinV2 maintained stronger robustness than ResNet-50, achieving AUC-ROC of 0.77 on DS2 compared with 0.68 for ResNet-50, and 0.76 on DS3 compared with 0.49 for ResNet-50. Ablation analyses further showed that multi-source data fusion and self-supervised pre-training substantially improved generalization, particularly for DS2 and DS3. Statistical evaluation using the Wilcoxon signed-rank test also supported the consistency of the proposed model’s improvements across paired train-test settings. These findings suggest that Multi-OCT-SelfNet-SwinV2 can learn more transferable OCT representations than conventional supervised baselines, making it a promising approach for robust AI-assisted retinal disease classification under data-limited and domain-shifted clinical conditions.

## 1 Introduction

In recent days, the artificial intelligence domain has witnessed a revolutionary breakthrough. However, in the medical field, a significant gap persists due to the scarcity of data. Machine learning models require extensive datasets for effective training, yet the medical domain faces constraints in this regard, primarily due to privacy concerns surrounding patient data. This scarcity poses a substantial challenge, hindering the progress and application of scalable medical AI solutions in healthcare.

To bridge this gap, our research addresses two key challenges. First, we focus on developing a robust machine learning classifier based on a transformer model to detect eye diseases from optical coherence tomography (OCT) images for AI-based eye care management. Second, we address the challenge of creating a machine learning model capable of learning from varied unlabeled data, making it useful in real-world situations with new and unseen data.

Age-related macular degeneration (AMD), along with other sight-threatening conditions such as diabetic macular edema (DME), choroidal neovascularization (CNV), and diabetic retinopathy (DR), ranks among the leading causes of irreversible blindness and vision impairment (VI) globally. VI affects nearly 2.2 billion people worldwide, with almost one billion cases potentially preventable through early diagnosis and intervention (World Health Organization, 2023). Therefore, it is critical to identify those who are at risk of developing the disease or seeing it progress, especially from the early stages to the more advanced stages, as prompt intervention can stop the disease’s progression or slow it down, ultimately preventing irreversible VI. Individuals at high risk for VI would greatly benefit from more frequent ophthalmic examinations, continuous monitoring, and prompt treatment (Scott and Bressler, 2013). Leveraging AI-based tools for early detection and continuous monitoring could significantly enhance our ability to identify at-risk individuals and intervene promptly, potentially saving countless individuals from needlessly suffering vision loss and impairment (Yi et al., 2009).

Automating diagnosis in ophthalmology has shown great promise with machine learning (ML) and deep learning (DL) techniques (Friberg et al., 2011; Alam et al., 2020b; Schmidt-Erfurth et al., 2018; Wang et al., 2016). However, the limited diversity of training datasets frequently impedes their effectiveness in the application of real-world clinical settings. When implementing these models in clinical settings, it is important to make a variety of datasets sourced from multiple institutions accessible to optimize their usefulness in clinical workflows. These datasets use different OCT image-capturing devices, cover a variety of demographics, and follow different protocols. By exposing our models to a range of datasets, we can increase their scalability, versatility, and adaptability, which will ultimately improve their performance and usefulness in real-world clinical scenarios.

This work improves retinal imaging detection and diagnosis, especially in automated ophthalmic diagnosis, by utilizing recent advances in large pre-trained transformer networks. This breakthrough performance of transformer models in NLP has raised great interest in the computer vision community. Since this architecture can learn the long-range dependencies within the data, it allows us to grasp the spatial and temporal relationships within images. By processing images as a sequence of patches, it can also capture the global context, which is very important in tasks such as image classification, object detection, and pose estimation. With the introduction of the first image-based transformer architecture, Vision Transformer (ViT) (Dosovitskiy et al., 2020), in 2020 by Alexey Dosovitskiy et al., it achieved state-of-the-art performance on image classification tasks, showcasing the potential applications of this transformer architecture in the field of computer vision, such as object detection, image segmentation, object tracking, etc. Later several transformer models were developed such as DeiT (Touvron et al., 2021), DETR (Carion et al., 2020), Swin Transformer (Liu et al., 2021), SwinV2 (Liu et al., 2022), VissionLLM (Wang et al., 2023), for the computer vision domain. However, employing a transformer architecture requires a significant amount of computational resources, posing a challenge for communities with limited computational infrastructure. Moreover, a large dataset is crucial for effective training, creating problems in cases where such extensive datasets are unavailable. Despite these challenges, researchers are actively working on solutions to address these issues through continuous advancements in hardware, the development of efficient algorithms, the implementation of data augmentation techniques, and the integration of synthetic data.

Inspired by masked autoencoders (He et al., 2022), we have developed a large-scale self-supervised model with random masking, utilizing a variation of transformer models, the SwinV2 (Liu et al., 2022) backbone, which is specifically for the classification of retinal diseases from optical coherence tomography (OCT) images. Our SwinV2-based classifier leverages the transformer architecture, a foundational component also employed in Large Language Models. Its attention mechanism enables the model to dynamically determine the relative importance of various input data regions, allowing it to capture complex patterns and relationships in the data. Similar to how LLMs process and comprehend textual data by concentrating on context and semantic relationships, SwinV2 performs well in visual tasks by paying attention to relevant image regions, which improves the accuracy of feature extraction and classification. The self-attention mechanism is applied to image patches by the SwinV2 model. SwinV2 is able to model global and local features more effectively by treating images as sequences of patches, in a manner similar to how text is processed as sequences of words or tokens. This leads to enhanced performance in image classification tasks. The integration of SwinV2 in our OCT image classification task demonstrates the versatility of transformer architectures beyond natural language processing. Notably, our focus extends to the multi-class classification task, encompassing the discrimination of normal cases from those presenting with AMD, choroidal neovascularization (CNV), diabetic macular edema (DME), and diabetic retinopathy (DR). Expanding on our earlier study, “OCT-SelfNet: A Self-Supervised Framework with Multi-Modal Datasets for Generalized and Robust Retinal Disease Detection” (Jannat et al., 2025), which concentrated solely on binary classification, this work extends the scope to a multi-class problem. Through the utilization of self-supervised learning (SSL), our model aims to alleviate the necessity for extensive manual annotations by experts, thereby reducing workload and facilitating broader clinical AI applications within retinal imaging data. Importantly, our model exhibits the capability to learn versatile and generalizable features from unlabeled retinal OCT datasets, a critical aspect for developing AI systems requiring fewer labeled examples to adapt to diverse diagnostic tasks.

Our study stands in contrast to previous research (Leandro et al., 2023; Tsuji et al., 2020; Awais et al., 2017; Lee et al., 2017), which have primarily focused on the analysis of singular datasets in isolation. In this methodology, the training centers on individual datasets, where models are trained on specific segments and then evaluated on the remainder. However, this type of framework presents challenges, particularly in practical clinical settings with limited dataset sizes. Deep learning models require larger datasets for effective training, and smaller datasets often result in poorer accuracy. Moreover, deploying a model trained on one clinical dataset to another setting is problematic due to variations in device settings and environmental factors. To handle these challenges, we investigate a more intricate method by examining the complexities posed by domain adaptation across multiple datasets. We integrate several OCT datasets—DS1, DS2, and DS3—sourced from three distinct studies conducted by Kermany et al. (2018), Srinivasan et al. (2014), Li et al. (2020), respectively.

This approach was motivated by several key considerations. First, integrating multiple datasets will allow the pre-trained model to learn from a more diverse dataset with different clinical settings, capturing a broader spectrum of imaging conditions. This diversity will help to develop a robust model that will generalize well across unseen patient populations and clinical settings. Second, the combined dataset will increase the amount of available training data. A larger dataset enables more effective training, as the model can be exposed to a wider variety of examples, reducing the risk of overfitting. Third, pre-training the model on this comprehensive dataset will allow it to leverage transfer learning effectively. The weights learned during the pre-training stage will serve as a strong foundation for the classifier network during fine-tuning. By starting with a model already familiar with a wide range of visual features, we can achieve better performance with less training data specific to our final task, improving both training efficiency and final accuracy. This comprehensive fusion of datasets allows us to construct a unified dataset, leveraging insights and data from various clinical settings to develop a more holistic understanding of domain adaptation complexities.

As a result of our approach, our model’s ability to generalize to novel, unseen data is enhanced. This proves especially beneficial in situations where access to extensive datasets is limited. Within the self-supervised pre-training phase, the model undergoes the training and validation processes on a combined large dataset. During this stage, we leverage the combined training and validation datasets from DS1, DS2, and DS3 to ensure comprehensive learning. Following pre-training, the model proceeds to the fine-tuning phase, where it is individually fine-tuned on each dataset. This fine-tuning process allows the model to adapt its learned representations to the specific characteristics of each dataset. Subsequently, the model is cross-evaluated across all test sets to assess its robustness and effectiveness across different datasets.

**FIGURE 1:**
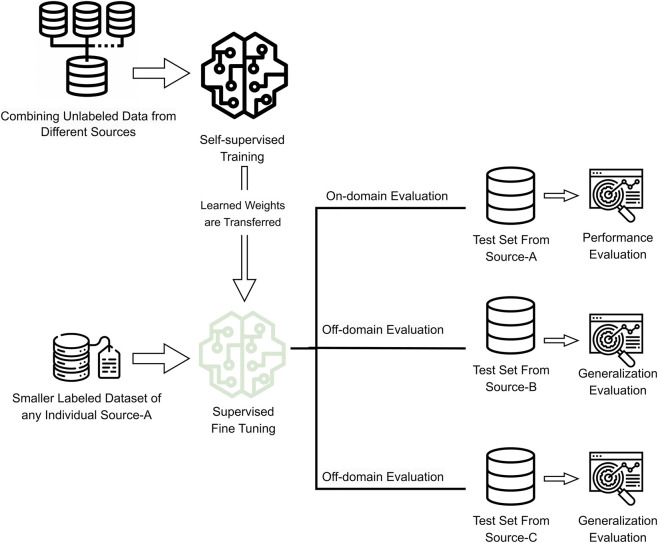
A graphical depiction of our methodology, consolidating fundamental concepts and procedural steps.

Our proposed method follows a comprehensive process that encompasses several key stages. Initially, the data fusion process occurs during the self-supervised pre-training phase, where information from multiple datasets is integrated to enhance model understanding and performance. Subsequently, fine-tuning is conducted on individual datasets to tailor the model to specific domain characteristics and optimize its performance further. Following fine-tuning, evaluation takes place on respective test sets, providing on-domain assessment measurements. Additionally, the model’s generalization capability is evaluated on other test sets for off-domain evaluation to measure its performance across diverse datasets and scenarios, thereby assessing its robustness and generalization capability. This process as depicted in Figure 1, illustrates the systematic approach employed in our methodology.

Comparative analysis against the baseline models, ResNet-50 and SwinV2, offers valuable insights into the performance of our proposed framework, Multi-OCT-SelfNet. Utilizing ResNet-50 and SwinV2 as a benchmark, we trained it on individual datasets and conducted cross-evaluation across all test sets to establish a reference point for comparison. Through an ablation study, we consistently observed the superiority of our Multi-OCT-SelfNet framework. AUC-ROC (Area Under the Receiver Operating Characteristic curve) and AUC-PR (Area Under the Precision-Recall curve) values are specifically used to measure performance. When comparing our suggested framework to the baseline ResNet-50 model, these metrics provide strong indications of its efficacy and dependability.

The primary contributions of this paper are outlined as follows:

- This work presents an approach of combining multiple datasets and showcases its efficacy in significantly improving classification performance on unseen datasets, while also demonstrating robust domain generalization capabilities.
- This paper introduces a two-phase methodology: firstly, employing a SwinV2-based masked autoencoder during pretraining, followed by a fine-tuning stage classifier for the classification of retinal diseases, specifically designed for Optical Coherence Tomography (OCT) use cases and multiclass classification tasks.
- The extensive evaluation and ablation studies conducted in this paper illustrate the robustness and generalizability of the proposed approach. Remarkably, even across different test sets, this method exhibits improved performance without additional fine-tuning. Such findings have promising implications for integration into real-world clinical settings.

## 2 Related works

In recent years, computer vision has emerged as an important tool in medical imaging, facilitating advanced diagnostics, treatment planning, and disease monitoring. The integration of deep learning (DL) techniques has particularly revolutionized this fieldby automating tasks such as image classification, segmentation, and disease diagnosis, thereby revolutionizing medical image analysis. Concurrently, transformer networks, originally designed for natural language processing, have shown promising potential, broadening the scope of applications within medical imaging. This section explores the latest developments and research efforts in leveraging DL and transformer networks for medical image analysis, with an emphasis on the contributions and advances made by them.

The field of computer vision has undergone a significant transformation due to the evolution of deep learning (DL), which began with the introduction of AlexNet Krizhevsky et al. (2012) in 2012. Serving as one of the first deep convolutional neural network (CNN) models, AlexNet’s success in the ImageNet was a major turning point in computer vision methodologies, transitioning from traditional methods to automated DL techniques. Subsequently, numerous backbone models like VGG Simonyan and Zisserman (2014), ResNet He et al. (2015), and Inception Szegedy et al. (2015) have been developed, further advancing image analysis capabilities. These advancements extended beyond traditional computer vision, impacting medical domains by demonstrating effectiveness in tasks such as medical image classification (Shazia et al., 2021; Krishnapriya and Karuna, 2023; Srinivas et al., 2022; Bressem et al., 2020; Yang et al., 2021; Choi et al., 2021; Le et al., 2020; Alam et al., 2020a). Notably, DL techniques have found significant application in the classification of optical coherence tomography (OCT) images, particularly in diagnosing conditions like age-related macular degeneration (AMD), choroidal neovascularization (CNV), and diabetic macular edema (DME). Studies such as those referenced by Tsuji et al. (2020) and Lee et al. (2017) have underscored the remarkable accuracy and effectiveness of DL in distinguishing abnormal OCT images from normal ones, indicating the potential for automated screening and the development of computer-aided diagnostic tools. However, DL algorithms, particularly CNNs, need substantial amounts of training data, which can be challenging to obtain in medical imaging, where data scarcity is common. Consequently, techniques such as transfer learning and domain adaptation have become essential for leveraging knowledge from source tasks to enhance performance in target tasks, addressing the data scarcity issue.

In recent advancements within medical image analysis, transformer models have emerged as a promising avenue for enhancing diagnostic accuracy and efficiency. Originally developed for natural language processing tasks, transformers have been adapted to handle the complex spatial relationships present in medical images. In 2017 (Vaswani et al., 2017), the transformer model was first introduced for the NLP task which leverages the self-attention mechanism to learn the contextual relationship among words within a sentence. Drawing inspiration from this concept, the vision transformer (ViT) (Dosovitskiy et al., 2020) utilizes multi-head attention mechanisms to understand the contextual relationships among image pixels. This design excels in learning long-range dependencies in data, enhancing its ability to interpret spatial and temporal aspects of images. By treating images as sequences of patches, ViT effectively comprehends the overall context, a crucial factor in image classification, object detection, and pose estimation. The introduction of ViT marks a significant milestone, setting new benchmarks in image classification and showcasing its immense potential in various computer vision applications, including medical image analysis. The application of transformer models in medical imaging, particularly in ophthalmology, has been extensively studied, as evidenced by works such as Ayana et al. (2023), Okolo et al. (2022), Alshammari et al. (2022), Wang et al. (2022), and Kihara et al. (2022). Notably, Wu et al. (2021) proposed a transformer-based approach tailored for fundus image analysis, wherein images are segmented into patches for sequential classification. This method has demonstrated remarkable performance in contrast to traditional convolutional neural networks (CNNs) across various metrics such as accuracy, specificity, precision, sensitivity, and quadratic weighted kappa score. The success of this method underscores the effectiveness of the applicability of attention mechanisms in diagnosing diabetic retinopathy. However, the adoption of a Vision Transformer (ViT) architecture poses challenges due to its heavy computational requirements, as highlighted by Islam (2022).

The development of a large language model, named BERT (Bidirectional Encoder Representations from Transformers) (Devlin et al., 2018), has revolutionized natural language processing tasks, demonstrating the power of self-supervised learning techniques. Inspired by their success in language understanding, researchers have begun exploring the application of these models in the computer vision domain. By leveraging the pre-trained representations learned from vast amounts of data, these models provide a novel way to address problems in medical image analysis, like limited labeled data scenarios. The subsequent introduction of Masked Image Modeling (MIM) marked a significant advancement in the field of self-supervised learning (SSL). MIM techniques, such as the masked autoencoder (MAE) introduced by He et al. (2022), focus on reconstructing masked portions of input data, allowing models to learn robust feature representations by capturing the underlying structure of visual data. Building upon this foundation, SimMIM (Xie et al., 2022) introduced a straightforward but successful method that directly predicts the pixel values of masked patches in images. These advancements highlight the potential of MIM-based approaches to address challenges in medical imaging, including data scarcity and the need for robust feature extraction, ultimately advancing diagnostic capabilities.

Several studies, including those by Fang et al. (2022), Qiu and Sun (2019), and Jing and Tian (2020), have underscored the growing importance of self-supervised learning (SSL) in the domain of ophthalmology-focused deep learning research. Tang et al. (2022) demonstrated the effectiveness of SSL in conjunction with the Swin UNETR architecture for analyzing 3D medical images, achieving state-of-the-art performance. Their findings highlight the potential of SSL to address challenges such as the scarcity of labeled data and the necessity for patient-specific diagnostic tools.

These works advance our understanding of how transformers and SSL can effectively extract meaningful information from large quantities of unlabeled data, ultimately advancing the capabilities of applicability of scalable medical AI solutions.

## 3 Methodology

In this work, we have taken a holistic approach by combining datasets sourced from three distinct origins. This process adds a wider range of information to the overall representation learning because each dataset contributes distinct sources. Combining a wide range of data sources improves performance and the model’s capacity to generalize to new data by giving it a deeper understanding of the data representation. We trained an SSL MAE network with SwinV2 as its backbone architecture by utilizing this combined dataset. This pre-trained weight makes a robust foundation for classifying retinal diseases effectively in the downstream tasks.

There are four essential stages in our proposed framework.

1. Data Fusion: Our study used three OCT image datasets, combining their training and validation sets for Self-Supervised Pre-training, followed by individual fine-tuning on each dataset to evaluate classification performance and generalization.
2. Self-Supervised Pre-training: In this initial stage of training, a self-supervised pre-training is conducted on a combined collection of unlabeled Optical Coherence Tomography (OCT) images, employing a transformer-based Masked Autoencoder (MAE) approach to extract detailed visual representations. Through this self-supervised learning process, the model gains an understanding of the structure and features within the multi-source OCT images. Subsequently, this learned weight is transferred to a supervised classifier model, leveraging the learned representations to improve the classification task.
3. Supervised Fine-tuning: Following the self-supervised pre-training phase, a supervised fine-tuning is conducted. This fine-tuning process aims to refine the model’s classification capabilities by leveraging the weights transferred from the pre-trained model. By exposing the model to labeled data and adjusting its parameters based on the specific task requirements, the fine-tuning stage further optimizes the model’s performance, enhancing its ability to accurately classify retinal diseases from OCT images.
4. Baseline Training: In our evaluation study, ResNet50 was used as the baseline model against which we compared the performance of our proposed model. By employing ResNet50 as a benchmark, we were able to assess the efficacy of our proposed approaches in improving our task.

### 3.1 Data fusion

**FIGURE 2:**
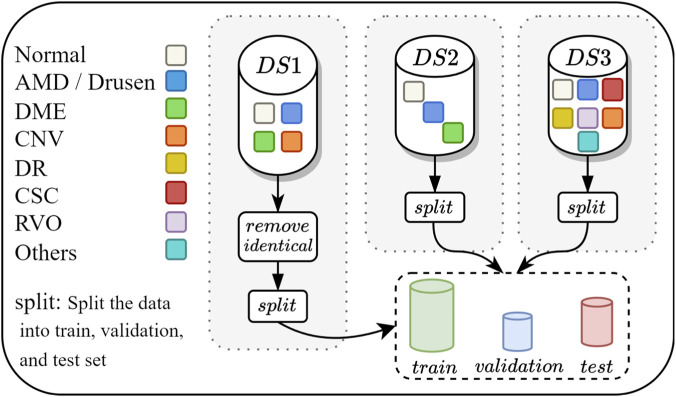
Illustration of the data combination process for the Self-Supervised Pre-training phase. Training and validation sets from three distinct Optical Coherence Tomography (OCT) datasets are merged to form a unified training and validation set, enhancing diversity and richness in the model’s representation learning.

For our study, we utilized three distinct datasets comprising Optical Coherence Tomography (OCT) images depicting various retinal diseases. Each dataset was partitioned into training, validation, and test sets. During the Self-Supervised Pre-training phase, we combined the training and validation sets from all three datasets into a unified training and validation set. This combination of data sources aims to enhance the diversity and richness of the training data, facilitating a broader representation learning of the model. By training on this combined dataset, the model acquired a more comprehensive representation of OCT images, which ultimately contributed to improving the generalization of unseen data. Subsequently, in the Supervised Fine-tuning stage, the classifier underwent fine-tuning on each training set and was evaluated on all test sets to assess classification performance and generalization capabilities across different datasets. In Figure 2, the overall data combination process is shown.

### 3.2 Self-supervised pre-training

Self-supervised learning (SSL) is a method where models learn from unlabeled data by understanding its structure. In this study, we used a technique called Masked Autoencoder (MAE), which masks parts of the input data randomly and trains the model to recreate the original by learning the representation of the input data. The MAE consists of two parts: an encoder and a decoder. We resized images to (224 × 224) and fed them through the encoder, which randomly masks 70% of the input image. For the encoder component, we explored the performance of two distinct networks—Swin and SwinV2—as backbone architectures, facilitating a comprehensive investigation into their effectiveness.

#### 3.2.1 Swin-based MAE

The encoder used in the Swin transformer-based Masked Autoencoder (MAE) is built with a Swin transformer backbone and has an embedding size of 96. The architecture of the Swin transformer has different numbers of layers at each stage (2, 2, 18, 2), which corresponds to the distribution of layers at each stage. The model employs shifted window attention mechanisms in each step to concentrate on local information within 4 × 4 patches, gradually constructing a global understanding through the connection of shifted windows. With each step, the number of attention heads—which is set to (6, 12, 24, 48)—doubles, allowing the model to attend to progressively finer details and identify hierarchical features in the input image. In the meantime, a more expressive representation is made possible during the decoding process by the decoder, which is constructed with an embedding size of 768.

The decoder network has a similar number of attention heads and layers as the encoder, along with Swin transformer layers that are set up to restore the spatial dimensions of the encoded features and a patch-expanding mechanism. This layer-wise design ensures a gradual reconstruction of the original image dimensions, facilitating effective decoding of the encoded representation acquired by the encoder.

#### 3.2.2 SwinV2-based MAE

For this task, we utilize a SwinV2-based Masked Autoencoder (MAE), capitalizing on the superior performance of the SwinV2 network. While retaining the Swin-based decoder, we used the SwinV2 for the encoder component to address challenges related to training stability, high-resolution processing, and data efficiency. The enhanced capabilities of SwinV2 align with our requirements, creating a balance between detail-oriented feature extraction and computational efficiency. Leveraging an embedding dimension of 96, depths configured as (2, 2, 6, 2), and attention heads ranging from (3, 6, 12, 24), this customized approach allowed the SwinV2-based MAEs to excel in capturing intricate details essential for our task.

For another experiment, we enhanced the Swin-V2 encoder to increase its dimensionality and depth, resulting in a more complex network, which we denote as SwinV2-large. In this architecture, we adjusted the embedding dimension to 196, while configuring depths as (4, 4, 4, 4), and attention heads as (6, 12, 24, 48).

### 3.3 Supervised fine-tuning

We added a classification head in place of the decoder in the classifier network. The classification head employed a linear layer to process the encoder’s features and produce class logits, which were then used for classification.

The linear layer consisted of three consecutive dense layers, each incorporating Rectified Linear Unit (ReLU) activation functions. The input image was gradually transformed into highly encoded features by these layers. The initial layer had an input size equal to the dimension of the positional embedding from the encoder, with an output size of 512. The subsequent layer refined these features, mapping them to a 256-dimensional space, followed by a final layer compressing them into a 128-dimensional feature vector.

The purpose of this hierarchical transformation was to prime the model for successful classification tasks by highlighting and reducing the amount of intrinsic discriminative features in the input. During training, the linear layer learns weights, and then class logits are transformed into class probabilities using the softmax function, allowing the model to predict classes accurately.

This methodology included fine-tuning one dataset, followed by assessing the model’s classification performance on the corresponding test set. Additionally, two separate test sets from distinct datasets were utilized to assess the model’s generalization and robustness. This iterative cross-data evaluation process was replicated across all three datasets, providing a thorough examination of the model’s adaptability to varying data sources.

The development of Multi-OCT-SelfNet, which combines fusion data with supervised fine-tuning and self-supervised pre-training techniques, offers a strong framework for the classification of retinal diseases in Optical Coherence Tomography (OCT) images. This framework attempts to enhance the model’s ability to generalize to new, unseen data by leveraging learned representations by combining the Masked Autoencoder (MAE) architecture with a subsequent classifier model. This holistic approach underscores the importance of utilizing fusion data with both self-supervised and supervised techniques to attain comprehensive and effective disease classification in OCT imaging.

**FIGURE 3:**
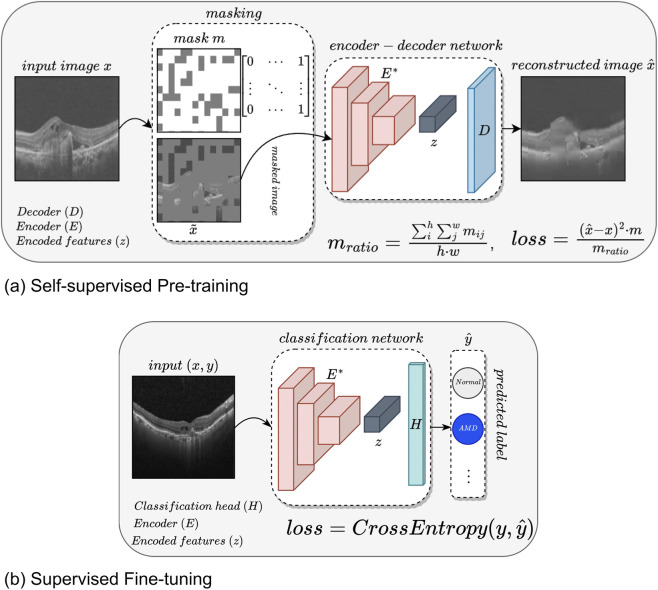
Overview of the Framework: **(a)** In the initial pre-training phase, the framework utilizes a masked image autoencoder as a self-supervised task to learn representations from unlabeled images. In this process, a random subset of image patches is masked and fed into the auto-encoder to reconstruct it. **(b)** In this phase, the pre-trained encoder from the first phase is employed along with a linear classifier for the classification task. The learned weights from the pre-training phase are transferred to the fine-tuning phase.

The overall framework is given in Figure 3, where the two-phase training approach is shown. Firstly, it utilizes a masked image autoencoder for self-supervised learning from unlabeled images. Then, in the second phase, the pre-trained encoder is combined with a linear classifier for classification tasks, transferring the learned weights from the initial phase. This strategy optimizes the model’s efficiency and effectiveness in handling classification tasks.

### 3.4 Baseline model

ResNet50 stands as a versatile solution for handling intricate tasks like classifying age-related macular degeneration (AMD) and diabetic retinopathy using Optical Coherence Tomography (OCT) images, showcasing its efficacy in medical imaging analysis. To evaluate the performance of our proposed method, we used ResNet50 as the baseline model, which is a conventional CNN network. The ResNet50 architecture comprises a 7 × seven kernel convolution and a max pooling layer, succeeded by a series of convolutional layers with varying sizes and numbers of kernels. With 50 convolutional layers, the network is then followed by average pooling and fully connected layers, with the number of nodes matching the classes for multi-class classification, employing softmax activation.

While the SwinV2-based MAE pre-training stage is computationally intensive, it is performed offline and only once. The downstream fine-tuning stage is comparatively lightweight and can be adapted to local clinical datasets with limited labeled data. Table 1 summarizes the network size and FLOPs (floating-point operations) of the proposed and baseline models. Although the proposed Multi-OCT-SelfNet-SwinV2 classifier has a moderately larger number of parameters than the baseline ResNet-50 (34.27 M vs. 23.5 M), its computational cost in terms of FLOPs remains comparable. This indicates that the proposed model introduces additional representational capacity without significantly increasing computational complexity, maintaining efficiency comparable to the ResNet-50 baseline.

**TABLE 1:** Network details.

Mode	Model	Model size (M)	FLOPS (G)
SSL-pre-training	Multi-OCT-SelfNet-SwinV2	33.79	5.62
Classifier	Resnet-50	23.5	4.1
	Multi-OCT-SelfNet-SwinV2	34.27	3.34

### 3.5 Loss function

For the pre-training stage, we have used a loss function, which only takes into account the pixels where the mask is active, and uses the mean squared error (MSE) between the predicted image and the original image. In Equation 1, the loss function is provided.

$$\frac{1}{m_{\mathrm{ratio}}}\times \frac{1}{N}\overset{N}{\underset{i=1}{\sum }}{\left(\bar{x_{i}}-x_{i}\right)}^{2}\times m_{i}$$ (1)

Here $\bar{x_{i}}$ is the predicted image, $x_{i}$ is the original input image, $m_{i}$ is the mask, $m_{\mathrm{ratio}}$ is the mask ratio, and N is the total number of samples.

The MSE is multiplied by the mask to calculate the loss on the pixels where the mask is active. The mask ratio indicates the percentage of the image that is masked. Since the mask is being used to focus only on specific areas of the image, the loss is calculated by dividing the mean squared error (MSE) by the mask ratio. This allows us to properly normalize the loss to the proportion of the image that is masked and scale the loss accordingly.

### 3.6 Datasets

#### 3.6.1 DS1

**FIGURE 4:**
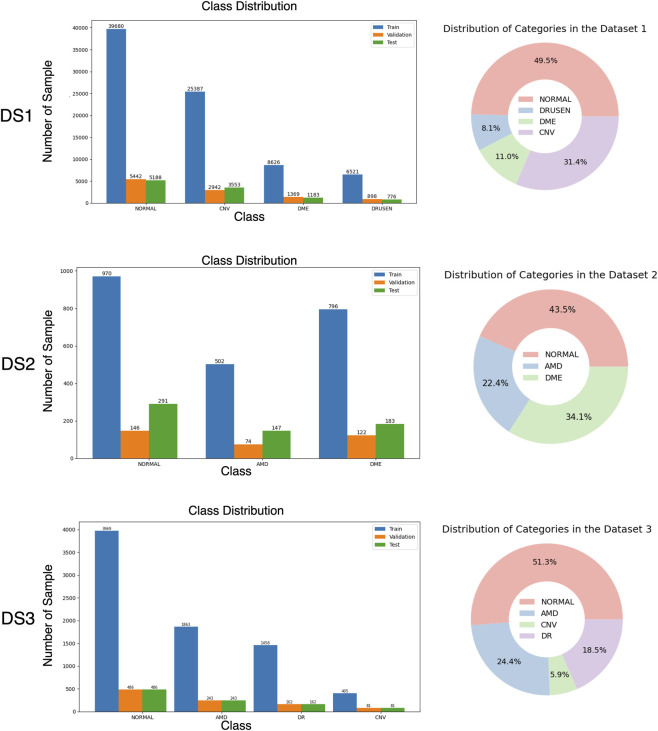
Distribution of retinal disease samples across three datasets: Grouped-bar diagrams show sample counts in training, validation, and test sets for each retinal disease category in datasets DS1, DS2, and DS3. The Donut charts display the overall percentage distribution per dataset.

This dataset comprises a total of 109,559 Optical Coherence Tomography (OCT) retinal images acquired using Spectralis OCT from Heidelberg Engineering, Germany (Kermany et al., 2018). These images are categorized into four classes: Normal, Choroidal Neovascularization (CNV), Diabetic Macular Edema (DME), and Drusen. Upon identifying identical images, as documented previously (Gholami et al., 2023), we followed their methodology to cleanse the dataset, resulting in 101,565 images. Subsequently, we reclassified Drusen images as Age-related Macular Degeneration (AMD). DS1 was then partitioned into training, testing, and validation sets using an 80%, 10%, and 10% ratio, respectively. The distribution of samples across each category within each set is illustrated in Figure 4.

#### 3.6.2 DS2

The DS2 dataset is acquired using Heidelberg Engineering Spectralis SD-OCT protocols approved by the Institutional Review Board (IRB) (Srinivasan et al., 2014), It consists of retinal images sourced from 45 subjects, distributed as follows: 15 normal subjects, 15 patients diagnosed with dry age-related macular degeneration (AMD), and 15 patients with diabetic macular edema (DME). To organize the dataset for training, validation, and testing purposes, we split the subjects accordingly: the first 10 subjects from each category were allocated to the training set, subjects 11 to 12 were assigned to the validation set, and subjects 13 to 15 were designated to the test set. Figure 4 shows the distribution of samples within each set, across each category.

#### 3.6.3 DS3

This dataset, comprising OCT images from 500 subjects, was obtained using two different fields of view: 3-mm and 6-mm. Each 3-mm file contains 304 scans per patient, while a 6-mm file contains 400 scans (Li et al., 2020). Our analysis focused on the slice images of the fovea (image numbers 160–240 from the 6-mm scans), capturing the most prominent retinal features, while peripheral retinal sections were deemed of limited significance for classification. This dataset comprises categories such as NORMAL, AMD, CNV, DR, OTHERS, RVO, and CSC. Given the relatively low number of samples in the RVO and CSC categories, we excluded them. The “OTHERS” category comprises diseases, including retinal detachment (RD), retinal hemorrhage (RH), and retinitis pigmentosa (RP), among others, which were also discarded due to their lack of particularity. All OCT images were captured using a spectral-domain OCT system with a center wavelength of 840 nm (RTVue-XR, Optovue, CA). Figure 4 provides an overview of the sample distribution across each category within each set.

In Figure 4, the distribution of retinal disease samples across training, validation, and test sets for each category in three datasets (DS1, DS2, and DS3) is shown. Grouped-bar diagrams depict the counts of samples in each category for training, validation, and test sets. Additionally, the donut charts illustrate the overall percentage distribution of samples for each dataset, providing a comprehensive view of the distribution of retinal disease samples in the study. Class presence and sample counts differ significantly; not all classes are present in all datasets in the same way. For example, although ‘NORMAL’ is present in all datasets, its frequency varies greatly: DS1 has a high count of 50,310 samples, DS3 has 4,941 samples, and DS2 has a significantly lower count of 1,407 samples. Similarly, the ‘AMD’ class exhibits varying degrees of representation, with 8,195 samples in DS1, 723 samples in DS2, and 2,349 samples in DS3. Such disparities extend further, with ‘DME’ being present in DS1 and DS2 but absent in DS3, while ‘DR’ finds exclusivity in DS3. These variations in class presence and sample counts highlight the complexities present in dataset composition and the implications for model development and evaluation. We primarily addressed this class imbalance complexities through self-supervised pre-training, multi-source data fusion, and the use of imbalance-robust evaluation metrics such as AUC-ROC, AUC-PR, and F1-score.

The model was fully trained with all classes found in datasets DS1, DS2, and DS3 during the self-supervised pre-training phase, which enabled it to fully understand the complexities of representation learning. Because of this inclusive training strategy, the network was able to capture a wide range of features and patterns that were present in the various classes. However, in the supervised fine-tuning stage, our focus narrowed to the classification tasks specifically targeting ‘NORMAL’ categories from the ‘AMD’, ‘DME’, ‘CNV’, and ‘DR’ classes. This two-stage training process, which includes thorough pre-training and specialized fine-tuning, strategically directs the model’s learning of its representation and maximizes its performance for the desired classification goal. This methodology helps the model to be optimally tuned to achieve the intended classification objectives by shifting from a general comprehension of all classes to a targeted refinement of the desired classification tasks.

## 4 Experiments

### 4.1 Implementation details

#### 4.1.1 Self-supervised pre-training implementation details

For this stage of self-supervised pre-training, all experiments were conducted using the computational power of the NVIDIA Tesla V100 graphical processing unit (GPU). Specific hyperparameters were selected to optimize model performance. The learning rate was set to $1.5\times 10^{-4}$ , and the Adam optimizer was employed with a weight decay of 0.05 (Loshchilov and Hutter, 2019). Additionally, the optimizer utilized $\beta_{1}$ and $\beta_{2}$ values of 0.9 and 0.95, respectively. Input data consisted of batches comprising 32 normalized images. Training proceeded for a total of 100 epochs. To ensure the most robust configuration, the model with the lowest validation loss was saved for subsequent fine-tuning iterations.

#### 4.1.2 Supervised fine-tuning implementation details

During the fine-tuning stage, similar to the training phase, experiments were conducted using the NVIDIA Tesla V100 GPU, and the hyperparameters were selected as follows: the learning rate was adjusted to $3\times 10^{-4}$ , and the Adam optimizer was employed with a weight decay of $1\times 10^{-6}$ . The optimizer utilized $\beta_{1}$ and $\beta_{2}$ values of 0.9 and 0.99, respectively. As the loss function, the categorical cross-entropy loss is employed. The training process involved the application of multiple data augmentation techniques, such as random resized crop, random horizontal flip, color jitter, random grayscale, and ImageNet normalization, to improve the robustness of the model. Training extended over 100 epochs, with early stopping criteria implemented using a patience of 10 epochs. The model with the highest validation accuracy was saved for subsequent testing.

All OCT images across datasets were processed using a consistent preprocessing pipeline to minimize potential confounding factors during fusion learning. Specifically, images were resized to a uniform resolution (224 × 224) and normalized using ImageNet-based intensity normalization parameters. No dataset-specific intensity scaling or scanner-dependent preprocessing was applied. While scanner-specific artifacts may inherently exist due to differences in acquisition devices, our use of a unified preprocessing strategy, combined with self-supervised pre-training and data fusion, was intended to encourage the model to learn robust and transferable representations across heterogeneous datasets. Python 3.10.9, Pytorch 1.12.1, and CUDA 11.2 were used to implement the code.

#### 4.1.3 Baseline model: ResNet-50 implementation details

During the experiments with the baseline model, an NVIDIA GeForce RTX 3060 Ti GPU was used. The learning rate was set to $3\times 10^{-4}$ , accompanied by a weight decay of $10^{-6}$ , a batch size of 24, and the utilization of the Adam optimizer with decoupled weight decay (Loshchilov and Hutter, 2019). Momentum and adaptive learning rate scaling were set by $\beta_{1}$ and $\beta_{2}$ values of 0.9 and 0.999, respectively. Training proceeded for a maximum of 100 epochs, with early stopping criteria based on validation loss, incorporating a patience of 10 epochs. To enhance the model’s robustness and generalization capabilities, various data augmentation techniques were employed, including random rotation, horizontal flip, color jittering, Gaussian blurring, and elastic transform.

### 4.2 Evaluation metrics

As illustrated in Figure 4, all datasets exhibit a substantial class imbalance, with a predominance of ‘NORMAL’ cases. Given this, relying solely on accuracy can be misleading. To mitigate this issue, we use AUC-ROC as the primary evaluation measure, as it effectively provides a more robust evaluation by considering the trade-off between true positive and false positive rates across different thresholds. Additionally, we incorporate accuracy, AUC-PR, and F1-score to offer a comprehensive evaluation. To obtain a more rigorous statistical assessment of the results, we also employed the Wilcoxon signed-rank test.

#### 4.2.1 Accuracy

The number of accurate predictions made by the model is known as accuracy, and it is the proportion of correct predictions among all predictions. The accuracy formula is provided by Equation 2.

$$Accuracy=\frac{TP+TN}{TP+TN+FP+FN}$$ (2)

Here, TP = True Positives, TN = True Negatives, FP = False Positives, and FN = False Negatives. Model *n* with accuracy scores *A_1_, A_2_, … , A_n_* is shown in Equation 4:

#### 4.2.2 AUC-ROC

One important metric for assessing the performance of the classifier is the Area Under the Receiver Operating Characteristic curve (AUC-ROC). Plotting each class’s true positive rate against its false positive rate across a range of threshold values creates the ROC curve. The area under this curve, or AUC-ROC, gives a thorough overview of the model’s performance in all classes and threshold settings. A higher AUC-ROC signifies better discrimination ability among the different classes, indicating superior classifier performance.

#### 4.2.3 AUC-PR

The Area Under the Precision-Recall curve (AUC-PR), a performance metric for classifier evaluation, is comparable to AUC-ROC. By plotting precision against recall across different threshold values, the PR curve emphasizes the trade-off between recall and precision. The AUC-PR quantifies the overall performance of the model across various threshold settings by measuring the area under this curve. A higher AUC-PR score indicates better classifier performance.

#### 4.2.4 F1-Score

Another performance evaluation metric that accounts for both recall and precision is the F1-Score. This metric is particularly helpful when dealing with data imbalances. By incorporating both precision and recall, the F1-Score provides a comprehensive evaluation of a classifier’s effectiveness. This metric is especially suitable in situations where accurately capturing both positive and negative instances is critical for decision-making and model evaluation.

Equation 3 is used to calculate the F1-Score.

$$F1Score=\frac{2*Precision*Recall}{Precision+Recall}$$ (3)

#### 4.2.5 Penalty-based performance index

To provide a quantitative assessment of the model’s generalization performance, we created the Penalty-Based Performance Index to evaluate the models’ performance across all test sets. This method calculates a penalty for each score by subtracting it from 1, representing the error rate. The scores considered for this calculation include accuracy, AUC-ROC, F1-score, and AUC-PR, encompassing various aspects of model performance. The average penalty for each model is then computed, indicating the overall error tendency which represents, on average, how much the model’s score deviates from a perfect score across all test sets. Finally, these average penalties are transformed into a scale of 1–100 to obtain a performance score for each model. A lower score indicates better performance, which means the model’s accuracy is closer to a perfect score with minimal variance across the test sets.

The formula for calculating the Penalty-Based Performance Index for Model 1, Model 2, … , Model n with accuracy scores $A_{1},A_{2},\ldots ,A_{n}$ is as follows:

$$Performance\;Index_{x}=\frac{\sum_{i=1}^{n}\left(1-A_{i}\right)}{n}\times 100$$ (4)

This evaluation method provides a quantitative measure of generalization performance by estimating how far each metric deviates from a perfect score across multiple test sets.

#### 4.2.6 Wilcoxon signed-rank test

The Wilcoxon signed-rank test is a non-parametric statistical test used to compare two related groups. It is useful when the same experimental settings are evaluated using two different methods, such as two classification models tested on the same datasets. For two paired sets of results, the difference between each pair is first calculated. The test then checks whether these differences are consistently positive or negative. If one method performs better in most paired comparisons, the Wilcoxon signed-rank test can determine whether this improvement is statistically significant. In this study, the test was used to compare model performance under the same train-test settings. This test was applied to paired summary metrics across matched train-test settings. A p -value was calculated for each comparison. If the p -value was less than 0.05, the difference between the two methods was considered statistically significant.

### 4.3 Self-supervised pre-training result

**FIGURE 5:**
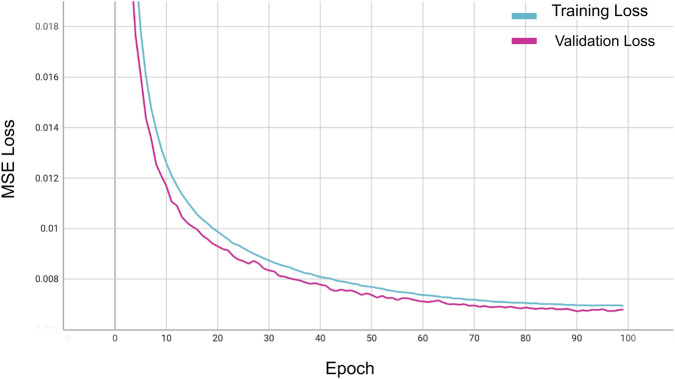
The training and validation MSE Loss curves of Multi-OCT-SelfNet with SwinV2 backbone, trained with the combined dataset.

**FIGURE 6:**
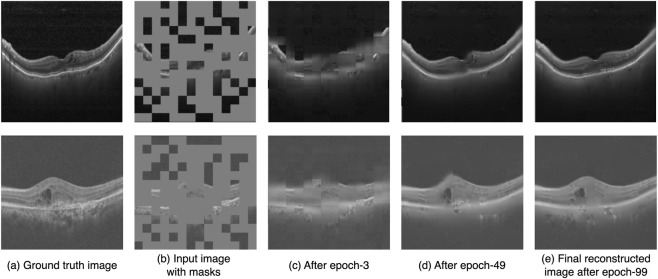
The progression results of the Multi-OCT-SelfNet-SwinV2 model on sample validation images across various epochs illustrate its learning process in reconstructing input images. From left to right, **(a)** is the corresponding ground truth image, **(b)** is the masked image input, and **(c–e)** is the reconstructed images in different epochs.

In our ablation study, we conducted pre-training for 100 epochs on three transformer-based networks. One network utilized the Swin architecture as its backbone, while the other two employed distinct variations of SwinV2 as backbones—referred to as SwinV2 and SwinV2-large, respectively. The objective was to assess the efficacy of these models and their performance in subsequent tasks. In particular, after 100 training epochs, the SwinV2-based MAE demonstrated remarkable proficiency, obtaining a mean squared error (MSE) loss of 0.007 as shown in Figure 5. Moreover, Figure 6 provides a detailed visualization of the masked image input and the corresponding image reconstruction from the SwinV2-based MAE at various epochs, offering insights into the model’s learning dynamics throughout the training process. While the reconstructed images may display imperfections, our project prioritized the capture of intricate image structures and patterns over flawless reconstructions. Consequently, in subsequent tasks, we employed these pre-trained weights, capitalizing on their learned representations, rather than initializing the classifiers with random weights. By applying the learned knowledge encoded in the pre-trained weights, this method allowed us to take advantage of the benefits of the transfer learning approach, facilitating enhanced performance and accelerated convergence in downstream tasks.

### 4.4 Supervised fine-tuning result

#### 4.4.1 Performance comparison with different encoder networks

**FIGURE 7:**
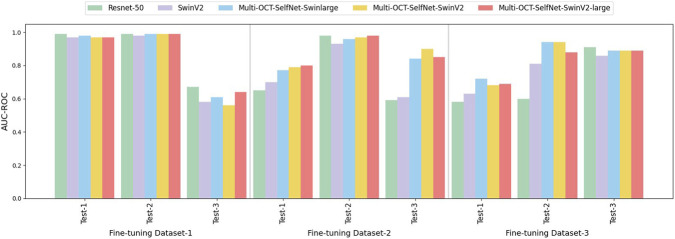
Comparison of AUC-ROC scores for different classifiers across three datasets and test sets. Each group represents AUC-ROC scores for classifiers within a specific dataset and test set combination, highlighting performance variations.

The performance comparison of different encoder networks is presented in Table 2 and Figure 7. The results show that the proposed Multi-OCT-SelfNet-SwinV2 model provides stronger and more stable performance than both baseline ResNet-50 and the traditional SwinV2 model, particularly when the training data are limited or when the model is evaluated on unseen datasets. This indicates that the proposed self-supervised pre-training and multi-dataset fusion strategy enables the SwinV2 encoder to learn more transferable OCT image representations than supervised training alone.

**TABLE 2:** Analyzing the performance with different encoder networks: Comparison of our framework with baseline methods (ResNet-50 and SwinV2) on the test sets from three datasets, evaluating multi-class classification performance in terms of accuracy, AUC-ROC, AUC-PR, and F1-score.

Dataset	Classifier name	AUC-ROC			Accuracy			AUC-PR			F1-score		
		Test-1	Test-2	Test-3	Test-1	Test-2	Test-3	Test-1	Test-2	Test-3	Test-1	Test-2	Test-3
DS1	Resnet-50-multi	0.99	0.99	0.67	0.95	0.92	0.24	0.96	0.99	0.53	0.95	0.92	0.25
	SwinV2	0.97	0.98	0.58	0.90	0.84	0.40	0.87	0.97	0.42	0.89	0.89	0.80
	Multi-OCT-SelfNet-swinlarge	0.98	0.99	0.61	0.91	0.92	0.41	0.89	0.99	0.43	0.90	0.94	0.54
	Multi-OCT-SelfNet-SwinV2	0.97	0.99	0.56	0.90	0.86	0.46	0.89	0.98	0.42	0.90	0.91	0.87
	Multi-OCT-SelfNet-SwinV2-large	0.97	0.99	0.64	0.91	0.91	0.40	0.90	0.99	0.44	0.91	0.93	0.56
DS2	Resnet-50-multi	0.65	0.98	0.59	0.29	0.87	0	0.39	0.95	0.57	0.31	0.87	0
	SwinV2	0.70	0.93	0.61	0.57	0.79	0.39	0.47	0.85	0.37	0.61	0.79	0.52
	Multi-OCT-SelfNet-swinlarge	0.77	0.96	0.84	0.62	0.88	0.45	0.54	0.92	0.64	0.65	0.87	0.59
	Multi-OCT-SelfNet-SwinV2	0.79	0.97	0.90	0.65	0.86	0.45	0.58	0.94	0.70	0.68	0.86	0.54
	Multi-OCT-SelfNet-SwinV2-large	0.80	0.98	0.85	0.68	0.93	0.28	0.61	0.97	0.63	0.70	0.93	0.73
DS3	Resnet-50-multi	0.58	0.60	0.91	0.55	0.66	0.86	0.45	0.82	0.74	0.39	0.53	0.82
	SwinV2	0.63	0.81	0.86	0.46	0.63	0.80	0.45	0.66	0.70	0.45	0.58	0.76
	Multi-OCT-SelfNet-swinlarge	0.72	0.94	0.89	0.51	0.86	0.88	0.52	0.90	0.85	0.49	0.82	0.85
	Multi-OCT-SelfNet-SwinV2	0.68	0.94	0.89	0.45	0.84	0.86	0.49	0.93	0.75	0.49	0.84	0.83
	Multi-OCT-SelfNet-SwinV2-large	0.69	0.88	0.89	0.52	0.79	0.87	0.49	0.85	0.79	0.51	0.71	0.85

For DS1, where a large number of training samples are available, all models achieved strong on-domain performance. ResNet-50, SwinV2, and Multi-OCT-SelfNet-SwinV2 produced comparable AUC-ROC values on Test-1 and Test-2. However, the advantage of the proposed method becomes more visible in the F1-score, especially on the cross-dataset Test-3 evaluation. When trained on DS1 and tested on Test-3, ResNet-50 achieved an F1-score of 0.25 and traditional SwinV2 achieved 0.80, whereas Multi-OCT-SelfNet-SwinV2 reached 0.87. This improvement suggests that the proposed framework better preserves class-level discrimination when applied to data from a different domain.

The benefit of Multi-OCT-SelfNet-SwinV2 is more evident on DS2, which contains fewer training samples. When trained on DS2 and evaluated on Test-3, ResNet-50 achieved an AUC-ROC of 0.59 and traditional SwinV2 achieved 0.61, while Multi-OCT-SelfNet-SwinV2 reached 0.90. This corresponds to an absolute improvement of 0.31 over ResNet-50 and 0.29 over traditional SwinV2. A similar trend is observed for AUC-PR, where the proposed model achieved 0.70 compared with 0.57 for ResNet-50 and 0.37 for traditional SwinV2. These results demonstrate that the proposed model is more robust in low-data settings and is better able to generalize to unseen OCT distributions.

For DS3, Multi-OCT-SelfNet-SwinV2 also showed stronger cross-dataset generalization. When trained on DS3 and tested on Test-2, the proposed model achieved an AUC-ROC of 0.94, compared with 0.60 for ResNet-50 and 0.81 for traditional SwinV2. The F1-score also improved substantially, increasing from 0.53 for ResNet-50 and 0.58 for traditional SwinV2 to 0.84 for Multi-OCT-SelfNet-SwinV2. On the DS3 on-domain test set, the proposed model maintained competitive AUC-ROC while improving F1-score compared with the traditional SwinV2 model, indicating better balance between sensitivity and precision across classes.

Figure 7 further supports these observations by showing that Multi-OCT-SelfNet-SwinV2 achieves consistently higher AUC-ROC values in the most challenging cross-dataset settings, especially for DS2 and DS3. The largest improvement is observed when training on DS2 and testing on Test-3, where the proposed model clearly outperforms both ResNet-50 and traditional SwinV2. This confirms that the improvement is not limited to on-domain testing, but extends to domain-shifted evaluation.

The penalty-based performance index in Table 3 provides additional evidence of improved generalization. Lower penalty values indicate better overall stability across the three test sets. For DS2, Multi-OCT-SelfNet-SwinV2 reduced the AUC-ROC penalty from 26.00 for ResNet-50 and 25.33 for traditional SwinV2 to 11.33. It also reduced the AUC-PR penalty from 36.33 to 43.67 to 26.00. For DS3, the proposed model reduced the AUC-ROC penalty from 30.33 for ResNet-50 and 23.33 for traditional SwinV2 to 16.33, while the F1-score penalty decreased from 42.00 to 40.33 to 28.00. These lower penalty scores confirm that Multi-OCT-SelfNet-SwinV2 generalizes more reliably across datasets.

**TABLE 3:** Analyzing model’s generalization performance on different encoder networks: Comparison of penalty-based performance scores across three test sets for different models and three datasets.

Dataset	Classifier name	P-index of AUC-ROC	P-index of accuracy	P-index of AUC-PR	P-index of F1-Score
DS1	Resnet-50-multi	**11.66**	29.6	**17.33**	29.33
	SwinV2	15.67	28.67	24.67	14
	Multi-OCT-SelfNet-swinlarge	14.00	**25.33**	23.0	20.66
	Multi-OCT-SelfNet-SwinV2	16.0	26.0	23.67	**10.67**
	Multi-OCT-SelfNet-SwinV2-large	13.33	25.99	22.33	19.99
DS2	Resnet-50-multi	26.0	61.33	36.33	60.66
	SwinV2	25.33	41.67	43.67	36
	OCT-SelfNet-swinlarge-multi	14.33	35.0	30.0	29.66
	OCT-SelfNet-SwinV2-multi	**11.33**	**34.67**	**26.00**	30.66
	OCT-SelfNet-SwinV2-large-multi	12.33	36.99	26.33	**21.33**
DS3	Resnet-50-multi	30.33	31.0	33.0	42.00
	SwinV2	23.33	37	39.67	40.33
	OCT-SelfNet-swinlarge-multi	**15.00**	**25.0**	**24.33**	**28.00**
	OCT-SelfNet-SwinV2-multi	16.33	28.33	27.67	**28.00**
	OCT-SelfNet-SwinV2-large-multi	18.00	27.33	28.99	31.0

Values shown in bold indicate the best performing result.

Overall, the results from Tables 2, 3, together with Figure 7, demonstrate that Multi-OCT-SelfNet-SwinV2 provides the most reliable performance among the compared models. While ResNet-50 and traditional SwinV2 can perform well when sufficient labeled data are available, their performance drops more noticeably under dataset shift and limited-data conditions. In contrast, the proposed framework benefits from self-supervised representation learning and multi-source OCT data fusion, leading to stronger transferability, improved F1-score, and lower generalization penalties across DS2 and DS3.

To provide statistical support for the observed improvements in Table 2, we performed a one-sided Wilcoxon signed-rank test using the paired metric values across the train-test combinations. The null hypothesis was that Multi-OCT-SelfNet-SwinV2 does not outperform the comparison model, while the alternative hypothesis was that Multi-OCT-SelfNet-SwinV2 achieves higher performance. Compared with traditional SwinV2, Multi-OCT-SelfNet-SwinV2 showed statistically significant improvements across all evaluation metrics, including AUC-ROC (p=0.0117) , accuracy (p=0.0078) , AUC-PR (p=0.0039) , and F1-score (p=0.0020) . When compared with ResNet-50, the strongest statistically significant improvement was observed for F1-score (p=0.0488) , while the pooled comparison across all Table 2 metrics was also significant (p=0.0075) . These results indicate that the proposed Multi-OCT-SelfNet-SwinV2 is not only numerically superior but also statistically more reliable, particularly compared with the traditional SwinV2 encoder. The improvement is especially meaningful because it is observed across repeated cross-dataset evaluations, supporting the claim that self-supervised pre-training with multi-source OCT data fusion improves generalization performance.

#### 4.4.2 Performance evaluation without data fusion during pre-training phase

To assess the impact of data fusion, we conducted an ablation study where self-supervised pre-training was performed on each dataset individually instead of using the fused dataset (Table 4). The results clearly demonstrate that data fusion significantly enhances performance, particularly for the smaller datasets DS2 and DS3.

**TABLE 4:** Analyzing the performance without data fusion during pre-training phase: Comparison of SwinV2-based classifier performance, where the encoder is pre-trained on individual training sets and the classifier is subsequently fine-tuned on the same training set, followed by evaluation on respective test sets.

Dataset name	Classifier name	AUC-ROC	Accuracy	AUC-PR	F1-score
DS1	Multi-OCT-SelfNet-SwinV2	0.97	0.89	0.88	0.90
DS2	Multi-OCT-SelfNet-SwinV2	0.89	0.74	0.80	0.74
DS3	Multi-OCT-SelfNet-SwinV2	0.67	0.58	0.35	0.47

Values shown in bold indicate the best performing result.

On DS2, without data fusion, the classifier achieved AUC-ROC of 0.89, Accuracy 0.74, AUC-PR 0.80, and F1-score 0.74. After incorporating data fusion (Table 2), performance improved to AUC-ROC 0.97, Accuracy 0.86, AUC-PR 0.94, and F1-score 0.86. This corresponds to a 9% gain in AUC-ROC, a 16% gain in accuracy, a 17.5% gain in AUC-PR, and a 16% gain in F1-score, showing the critical role of fusion in handling limited data scenarios.

On DS3, the performance gap was even larger. Without fusion, the model achieved only AUC-ROC 0.67, Accuracy 0.58, AUC-PR 0.35, and F1-score 0.47. With fusion, these metrics were improved to AUC-ROC 0.89 (+33%), Accuracy 0.86 (+48%), AUC-PR 0.75 (+114%), and F1-score 0.83 (+77%). This represents the most significant improvement, highlighting that fusion compensates for the smaller dataset size and higher variability of DS3.

In contrast, for DS1, which already has a large sample size, the effect of data fusion was modest. The model improved only slightly from Accuracy 0.89 → 0.91 (+2%), AUC-ROC 0.97 → 0.97 (no change), AUC-PR 0.88 → 0.89 (+1%), and F1-score 0.90 → 0.90 (no change). This indicates that DS1 had sufficient data to support robust representation learning even without fusion.

Overall, these findings confirm that data fusion contributes disproportionately to performance improvements in smaller datasets. For DS2 and DS3, data fusion enhanced AUC-ROC by up to 33%, accuracy by 48%, and F1-score by as much as 77%. In contrast, DS1 benefited only marginally due to its large sample size. Thus, data fusion is particularly impactful in scenarios where datasets are scarce or imbalanced, enabling robust cross-dataset generalization.

To statistically assess the impact of data fusion, we applied a one-sided Wilcoxon signed-rank test using the paired on-domain results from the three datasets. The model with data fusion was compared against the model without data fusion for each metric across DS1, DS2, and DS3. Although the per-metric tests showed consistent improvements after data fusion, the small number of paired observations limited statistical significance for individual metrics: AUC-ROC (p=0.250) , accuracy (p=0.125) , AUC-PR (p=0.125) , and F1-score (p=0.250) . However, when all paired metric values were jointly considered, the improvement with data fusion was statistically significant (p=0.001) . This indicates that data fusion provides a consistent overall performance benefit, especially for the smaller datasets DS2 and DS3, where the gains in accuracy, AUC-PR, and F1-score were substantially larger than those observed for DS1.

#### 4.4.3 Performance evaluation on the effect of self-supervised pre-training

**FIGURE 8:**
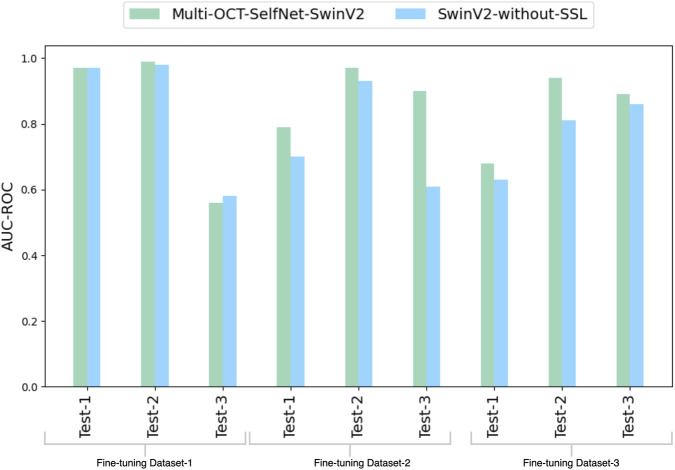
Comparison of AUC-ROC scores for multi-OCT-SelfNet-SwinV2 classifier without and with self-supervised pretraining phase across three datasets and test sets.

We further evaluated the contribution of self-supervised pre-training by comparing Multi-OCT-SelfNet-SwinV2 (with SSL) against a SwinV2 model trained from scratch (without SSL). The results (Table 5; Figure 8) clearly show that self-supervised pre-training substantially improves generalization across datasets, particularly for DS2 and DS3.

**TABLE 5:** Analyzing the impact of self-supervised pre-training: comparing our framework with SwinV2 classifier on test sets from three datasets. evaluation includes AUC-ROC, accuracy, AUC-PR, and F1-score for multi-class classification performance.

Dataset	Classifier name	AUC-ROC			Accuracy			AUC-PR			F1-score		
		Test1	Test2	Test3	Test1	Test2	Test3	Test1	Test2	Test3	Test1	Test2	Test3
DS1	Multi-OCT-SelfNet-SwinV2	**0.97**	**0.99**	0.56	**0.90**	**0.86**	**0.46**	**0.89**	**0.98**	**0.42**	**0.90**	**0.91**	**0.87**
	SwinV2-without-SSL	**0.97**	0.98	**0.58**	**0.90**	0.84	0.40	0.87	0.97	**0.42**	0.89	0.89	0.80
DS2	Multi-OCT-SelfNet-SwinV2	**0.79**	**0.97**	**0.90**	**0.65**	**0.86**	**0.45**	**0.58**	**0.94**	**0.70**	**0.68**	**0.86**	**0.54**
	SwinV2-without-SSL	0.70	0.93	0.61	0.57	0.79	0.39	0.47	0.85	0.37	0.61	0.79	0.52
DS3	Multi-OCT-SelfNet-SwinV2	**0.68**	**0.94**	**0.89**	0.45	**0.84**	**0.86**	**0.49**	**0.93**	**0.75**	**0.49**	**0.84**	**0.83**
	SwinV2-without-SSL	0.63	0.81	0.86	**0.46**	0.63	0.80	0.45	0.66	0.70	0.45	0.58	0.76

Values shown in bold indicate the best performing result.

On DS1, both models achieved similar on-domain results due to the dataset’s large size. With SSL, the model achieved AUC-ROC of 0.97, Accuracy 0.90, AUC-PR 0.89, and F1-score 0.90, while without SSL the scores were AUC-ROC 0.97, Accuracy 0.90, AUC-PR 0.87, and F1-score 0.89. The improvements were minor (1%–2%), confirming that DS1 provides sufficient labeled data to support strong supervised training, even without pre-training.

On DS2, the benefits of SSL were much more noticeable. With SSL, AUC-ROC improved from 0.70 (no SSL) to 0.79 (+13%), AUC-PR improved from 0.47 to 0.58 (+23%), and F1-score increased from 0.61 to 0.68 (+11%). Accuracy also rose from 0.57 to 0.65 (+14%). These results demonstrate that pre-training enables the model to extract transferable features from unlabeled data, significantly enhancing classification in smaller datasets.

On DS3, SSL yielded even larger gains. AUC-ROC improved from 0.63 → 0.68 (+8%), AUC-PR from 0.45 → 0.49 (+9%), and F1-score from 0.45 → 0.49 (+9%). Accuracy remained nearly unchanged from 0.46 → 0.45 (stable). While the absolute improvements seem modest, they consistently demonstrate that pre-training stabilizes learning and boosts performance across multiple metrics in limited-data conditions.

Figure 8 further highlights these trends: across nearly all dataset-test set combinations, the SSL-pretrained SwinV2 consistently outperformed its non-pretrained counterpart in terms of AUC-ROC. The gap was largest in DS2 and DS3 off-domain evaluations, confirming the crucial role of SSL in enhancing generalization.

The Penalty-Based Performance Index (Table 6) provides additional confirmation. On DS2, SSL reduced the AUC-ROC penalty from 25.33 (no SSL) to 11.33 (SSL), a 55% reduction, and lowered the accuracy penalty from 41.67 → 34.67 (−17%). Similarly, on DS3, SSL reduced the AUC-ROC penalty from 23.33 → 16.33 (−30%) and the F1-score penalty from 40.33 → 28.0 (−31%). These reductions highlight improved robustness and reduced error rates when using SSL.

**TABLE 6:** Analyzing model’s generalization performance with or without SSL pre-training: Comparing our proposed framework with SwinV2 network, which is not pre-trained with SSL, assessing penalty-based performance scores across three test sets for different datasets.

Dataset	Classifier name	P-index of AUC-ROC	P-index of accuracy	P-index of AUC-PR	P-index of F1-Score
DS1	Multi-OCT-SelfNet-SwinV2	16.0	**26.0**	**23.67**	**10.67**
	SwinV2-without-SSL	**15.67**	28.67	24.67	14.00
DS2	Multi-OCT-SelfNet-SwinV2	**11.33**	**34.67**	**26.00**	**30.66**
	SwinV2-without-SSL	25.33	41.67	43.67	36.00
DS3	Multi-OCT-SelfNet-SwinV2	**16.33**	**28.33**	**27.67**	**28.00**
	SwinV2-without-SSL	23.33	36.99	39.67	40.33

Values shown in bold indicate the best performing result.

In summary, while DS1 benefited only slightly from SSL due to its larger training set, DS2 and DS3 exhibited substantial improvements. Self-supervised pre-training led to 8%–23% increases in AUC-PR and F1-scores, and up to 55% reductions in penalty scores, underscoring its importance in achieving generalization and stability in smaller or imbalanced datasets.

To statistically verify the contribution of self-supervised pre-training, we applied a one-sided Wilcoxon signed-rank test using the paired summary metrics from Table 5. The SSL-pretrained Multi-OCT-SelfNet-SwinV2 was compared with SwinV2 without SSL under identical train-test settings. The SSL-pretrained model showed significant improvements in AUC-ROC (p=0.0117) , accuracy (p=0.0078) , AUC-PR (p=0.0039) , and F1-score (p=0.0020) . The pooled comparison across all metric values was also significant (p<0.001) . This supports the observation that SSL improves the stability and generalization capability of the SwinV2 encoder, especially for smaller and domain-shifted datasets.

#### 4.4.4 Performance comparison in limited data settings

**FIGURE 9:**
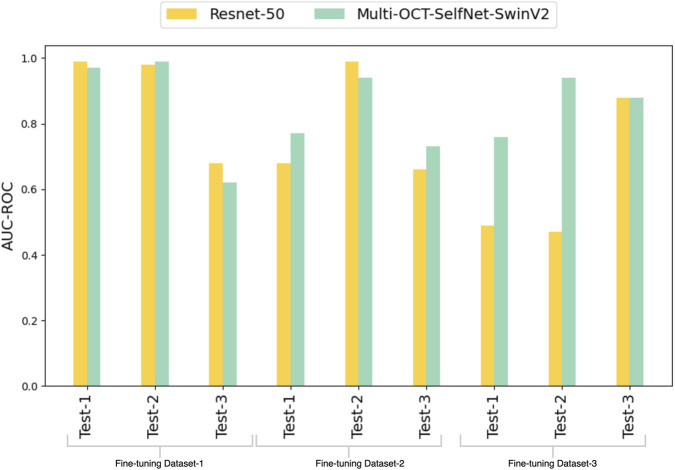
Comparison of AUC-ROC Scores Between Our Method (Multi-OCT-SelfNet-SwinV2) and Baseline (ResNet-50) on Test Sets from Three Datasets, when only 50% of Training Data has been used for Fine-Tuning.

To evaluate robustness under limited data availability, we fine-tuned both the baseline ResNet-50 and Multi-OCT-SelfNet-SwinV2 using only 50% of the training data from each dataset. The results (Table 7; Figure 9) show that while ResNet-50 suffered significant performance degradation, Multi-OCT-SelfNet-SwinV2 maintained strong accuracy and generalization, particularly on DS2 and DS3.

**TABLE 7:** Analyzing the performance in limited data settings: comparison of our work with the baseline methods on test sets from three datasets, using only 50% of the training set from each dataset in finetuning. The evaluation focuses on the classification AUC-ROC, accuracy, AUC-PR, and F1-score.

		AUC-ROC			Accuracy			AUC-PR			F1-score		
Dataset	Classifier name	Test1	Test2	Test3	Test1	Test2	Test3	Test1	Test2	Test3	Test1	Test2	Test3
DS1	Resnet-50	0.99	0.98	0.68	0.94	0.91	0.66	0.96	0.98	0.44	0.94	0.91	0.61
	Multi-OCT-SelfNet-SwinV2	0.97	0.99	0.62	0.90	0.89	0.46	0.89	0.98	0.44	0.90	0.93	0.61
DS2	Resnet-50	0.68	0.99	0.66	0.30	0.90	0.0	0.40	0.99	0.59	0.33	0.90	0.0
	Multi-OCT-SelfNet-SwinV2	0.77	0.94	0.73	0.70	0.83	0.41	0.56	0.90	0.48	0.71	0.83	0.62
DS3	Resnet-50	0.49	0.47	0.88	0.37	0.0	0.91	0.43	0.56	0.72	0.20	0.0	0.87
	Multi-OCT-SelfNet-SwinV2	0.76	0.94	0.88	0.47	0.80	0.81	0.56	0.91	0.73	0.49	0.83	0.78

On DS1, which contains ample samples, both models performed competitively. ResNet-50 achieved AUC-ROC of 0.99, Accuracy 0.94, AUC-PR 0.96, and F1-score 0.94, while Multi-OCT-SelfNet-SwinV2 achieved AUC-ROC of 0.97, Accuracy 0.90, AUC-PR 0.89, and F1-score 0.90. Here, ResNet-50 slightly outperformed Multi-OCT-SelfNet-SwinV2 (+2–5%), likely due to DS1’s large dataset size, which benefits supervised training.

On DS2, however, Multi-OCT-SelfNet-SwinV2 demonstrated clear superiority. ResNet-50 dropped to AUC-ROC 0.68, Accuracy 0.30, AUC-PR 0.40, and F1-score 0.33, showing a severe collapse in generalization. In contrast, Multi-OCT-SelfNet-SwinV2 achieved AUC-ROC 0.77 (+13%), Accuracy 0.70 (+133%), AUC-PR 0.56 (+40%), and F1-score 0.71 (+115%). These improvements highlight the strong advantage of pre-trained Multi-OCT-SelfNet-SwinV2 when training data is limited.

On DS3, the performance gap was even more pronounced. ResNet-50 achieved only AUC-ROC 0.49, Accuracy 0.37, AUC-PR 0.43, and F1-score 0.20, whereas Multi-OCT-SelfNet-SwinV2 reached AUC-ROC 0.76 (+55%), Accuracy 0.47 (+27%), AUC-PR 0.56 (+30%), and F1-score 0.49 (+145%). Notably, Multi-OCT-SelfNet-SwinV2 improved the F1-score by more than 2 × over ResNet-50, demonstrating superior classification under scarce training conditions.

Figure 9 visualizes these results, showing consistently higher AUC-ROC values for Multi-OCT-SelfNet-SwinV2 compared to ResNet-50 across DS2 and DS3 test sets, with the largest margin observed in DS3 (0.49 → 0.76, +55%).

The Penalty-Based Performance Index (Table 8) further confirmed these trends. On DS2, SwinV2 reduced the accuracy penalty from 60.0 (ResNet-50) to 35.33 (−41%), and the F1-score penalty from 59.0 → 28.0 (−53%). On DS3, the improvement was even greater: accuracy penalty decreased from 57.33 → 30.67 (−47%), and F1 penalty from 64.33 → 30.0 (−53%). These reductions illustrate stronger cross-dataset stability under limited training.

**TABLE 8:** Analyzing model’s generalization performance in limited data settings: Comparing our proposed framework with SwinV2 network with the baseline model on test sets from three datasets, using only 50% of the training set from each dataset in fine-tuning. Assessing penalty-based performance scores across three test sets for different datasets.

Dataset	Classifier name	P-index of AUC-ROC	P-index of accuracy	P-index of AUC-PR	P-index of F1-Score
DS1	Resnet-50	**11.67**	**16.33**	**20.67**	**18.00**
	Multi-OCT-SelfNet-SwinV2	14.00	25.0	23.0	18.67
DS2	Resnet-50	22.33	60.0	**34.0**	59.0
	Multi-OCT-SelfNet-SwinV2	**18.67**	**35.33**	35.33	**28.00**
DS3	Resnet-50	38.67	57.33	43.0	64.33
	Multi-OCT-SelfNet-SwinV2	**14.00**	**30.67**	**26.67**	**30.0**

Values shown in bold indicate the best performing result.

In summary, while ResNet-50 performed slightly better on the large-scale DS1, Multi-OCT-SelfNet-SwinV2 delivered better performance on DS2 and DS3. This demonstrates that our SSL-based framework is highly effective in scenarios where labeled training data is scarce, maintaining strong classification and generalization ability across domains.

To statistically evaluate the limited-data setting, we applied a one-sided Wilcoxon signed-rank test using the paired results from Table 7. The performance of Multi-OCT-SelfNet-SwinV2 was paired with the corresponding ResNet-50 result under the same train-test setting. For individual metrics, the improvements were not statistically significant at the p<0.05 level: AUC-ROC (p=0.125) , accuracy (p=0.258) , AUC-PR (p=0.234) , and F1-score (p=0.125) . This is expected because ResNet-50 remained competitive on DS1, where sufficient samples were still available even after reducing the training set. However, when all paired metric values were jointly considered, Multi-OCT-SelfNet-SwinV2 showed a statistically significant overall improvement over ResNet-50 (p=0.027) . This supports the observation that the proposed SSL-based framework provides more reliable performance under limited labeled data, particularly for the smaller and more challenging DS2 and DS3 datasets.

## 5 Future work

While our framework has shown significant potential in the automated classification of retinal diseases using OCT images, its complexity poses challenges in terms of interpretability. As we move forward, enhancing the transparency of our model is a key priority. By improving interpretability, we aim to build AI-based diagnostic tools that clinicians can trust and readily understand, ensuring that these models are not only powerful but also transparent in their operations. Additionally, we recognize the importance of continuous improvement in AI systems, particularly in the context of their application in diverse clinical environments. Our future work will focus on integrating a human-in-the-loop system to complement the model’s capabilities. By involving human expertise, we can enhance the model’s adaptability, enabling it to learn and improve from real-world feedback continuously. This approach will help mitigate issues related to cross-domain generalization, ensuring that our model maintains high performance across different clinical settings.

Inconsistent labeling or errors in the dataset can introduce biases and negatively affect the model’s performance, particularly when combining datasets from multiple sources. In this process, the domain experts will periodically review the model’s predictions, especially in examples where the model is uncertain. This iterative feedback loop will help to continuously refine label quality and enhance model accuracy by effectively managing labeling inconsistencies and errors in the dataset.

## 6 Conclusion

The proposed work aims to leverage innovative techniques such as multi-source data fusion, self-supervised learning, and transformer networks to overcome the challenges posed by limited data availability in retinal disease diagnosis. During the self-supervised phase, the model is pre-trained using publicly available datasets, ensuring that sensitive clinical data is not exposed. In the downstream classification task specific to individual clinical settings, the model is fine-tuned solely on the local dataset, eliminating the need to share any private data. This approach preserves data privacy while allowing the model to adapt effectively to diverse clinical environments. This framework will be beneficial in situations where access to extensive datasets is limited, offering a scalable and practical approach to implementing medical AI solutions. This proposed framework could be integrated into practical clinical workflows. Specifically, the self-supervised pre-training stage can be performed offline using publicly available or institutionally approved datasets, while the downstream fine-tuning stage can be adapted to local clinical data without requiring data sharing. This design supports privacy preservation and facilitates deployment across diverse clinical environments. The results of our study showcase the effectiveness of our proposed framework, Multi-OCT-SelfNet, which consistently surpasses the baseline performance. Our approach demonstrates superior performance scores across all datasets, particularly excelling with smaller datasets. Through meticulous experimentation, we’ve validated the efficacy of our methodology. The incorporation of data fusion and self-supervised pre-training significantly enhances performance, as evidenced by our ablation study. This highlights the significance of these components in improving both the resilience and accuracy of our model, thereby facilitating robust generalization.

## Data Availability

The datasets used in this study are publicly available: DS1 ‐ Kermany et al., 2018, DS2 ‐ Srinivasan et al., 2014, and DS3 - OCTA-500 is available at this link, https://ieee-dataport.org/open-access/octa-500.
